# Intestinal Anisakiasis presenting as a small bowel obstruction - Pitfalls in the application of ultrasound

**DOI:** 10.1186/2036-7902-4-S1-A8

**Published:** 2012-12-18

**Authors:** Masaaki Ogata

**Affiliations:** 1 Kobe City MC West Hospital, Emergency Medicine, Kobe, Japan

## Background

Intestinal Anisakiasis is a relatively rare disease, which is related to the ingestion of raw or undercooked fish. It often presents as acute abdomen, and may mimic acute appendicitis, peritonitis and small bowel obstruction. It may be explored by laparotomy, although it usually resolves with conservative treatments.

## Objectives

The purpose of the study is to evaluate the usefulness of ultrasound in the diagnosis of intestinal Anisakiasis presenting as a small bowel obstruction.

## Patients and methods

Seven patients with intestinal Anisakiasis presenting as a small bowel obstruction, who were admitted in the Kobe City Medical Center West Hospital between June 2009 and May 2012, were included in the study. The final diagnosis was made clinically on the basis of medical history, clinical pictures and imaging findings in addition to serology (specific IgE to Anisakis measured with Pharmacia CAP system). We retrospectively reviewed clinical features, ultrasonograms and CT images of the cases, and compared the efficacy of ultrasound with that of CT scan in the diagnosis of intestinal Anisakiasis.

## Results

All of the cases had a significant level of specific IgE to Anisakis. A present history of eating some kinds of raw fish was confirmed a couple of days before they presented to the emergency department with complaints of abdominal pain and/or distension. No past history of laparotomy was identified in 6 of them. Both ultrasound and CT scan showed the evidence of small bowel obstruction, showing dilated small bowel (7/7) and accumulation of peritoneal fluid (6/7). CT scan demonstrated segmental wall thickening and stenosis with submucosal edema distal to the dilated small bowel in all, but ultrasound demonstrated the finding in 4 of 7 cases.

## Conclusions

Ultrasound was useful in showing dilated small bowel and peritoneal fluid, but inferior to CT scan in demonstrating segmental intestinal edema causing a small bowel obstruction in cases of intestinal Anisakiasis.
